# Exploiting the ability of *Bacillus subtilis* to synthetize surfactin under oxygen limitation

**DOI:** 10.1007/s00253-026-13868-0

**Published:** 2026-05-14

**Authors:** Eric Hiller, Lea Rahel Tadele, Nikolina Miceta, Dominik Notz, Elvio Henrique Benatto Perino, Rudolf Hausmann

**Affiliations:** Department of Bioprocess Engineering, Institute of Food Science and Biotechnology, University of Hohenheim, Stuttgart, Germany

**Keywords:** Lipopeptide production, Oxygen-limited cultivation, Fed-batch fermentation, Bioreactor scale-up, Process kinetics, Nitrogen metabolism

## Abstract

**Abstract:**

Surfactin production by *Bacillus subtilis* is typically performed under aerobic conditions, requiring high aeration and agitation, which leads to mechanical stress for the cells and therefore promotes excessive foaming. As a process-oriented alternative to full aerobic operation, an aerobic to micro-aerobic switching strategy was developed that aims to decouple biomass formation from surfactin synthesis by controlling oxygen availability. Promoter activities relevant to nitrate respiration and surfactin biosynthesis were first characterised in shake flasks using transcriptional reporter strains and online monitoring of dissolved oxygen. The nitrite reductase promoter showed the strongest induction under oxygen-limited conditions and was used to construct an oxygen-responsive production strain in which the native P_*srfA*_ promoter was replaced to suppress surfactin formation during aerobic growth and shift production to the micro-aerobic phase. The switching concept was subsequently evaluated in 30-L stirred-tank bioreactor cultivations using stepwise reductions of dissolved oxygen setpoints combined with exponential glucose feeding and nitrate supplementation. The engineered strain *B. subtilis* MG19 enabled stable transitions into micro-aerobic operation without apparent loss of biomass, while a reference strain *B. subtilis* MG17 with the native regulation showed surfactin formation already during the aerobic phase and pronounced process disturbance after switching, characterised by glucose accumulation and a strong decrease in surfactin concentration. Overall, the study demonstrates oxygen switching as a scalable process engineering tool for controlling surfactin production phases and highlights key constraints for robust micro-aerobic bioreactor operation.

**Key points:**

• *Oxygen availability decoupled growth and surfactin formation.*

• *Micro-aerobic process control was evaluated in 30-L bioreactor cultivations.*

• *The engineered strain shifted surfactin production to oxygen-limited conditions.*

**Supplementary Information:**

The online version contains supplementary material available at 10.1007/s00253-026-13868-0.

## Introduction

Surfactin is a cyclic lipopeptide produced by *Bacillus subtilis* and is considered one of the most powerful biosurfactants known, due to its exceptionally low critical micelle concentration of about 15 mg/L and strong surface- and interfacial tension-reducing properties in low concentrations (Cooper et al. 1981; Hoffmann et al. 2021b; Maget-Dana & Ptak 1992). The chemical structure of surfactin consists of a peptide ring with seven amino acids connected to a β-hydroxy fatty acid chain with 12 to 19 carbon atoms (Arima et al. 1968). In addition to its physicochemical functionality, surfactin exhibits antiviral, anti-inflammatory, and hemolytic activities (Kracht et al. 1999; Vollenbroich et al. 1997; Zang et al. 2014). Its amphiphilic structure and broad bioactivity have therefore driven extensive research into efficient and scalable production processes (Hiller et al. 2024; Klausmann et al. 2021).

*B. subtilis* is a well-established microbial cell factory and a relevant host for surfactin production, benefiting from its genetic accessibility and robustness in cultivations (Hermann et al. 2025; Klausmann et al. 2021). Importantly, *B. subtilis* is a facultative anaerobe capable of adapting its metabolism to oxygen-limited conditions by utilizing alternative respiratory pathways, such as nitrate respiration (Bohin et al. 1976; Glaser et al. 1995; Nakano & Zuber 1998). This metabolic flexibility provides an opportunity to decouple biomass formation and secondary metabolite production through controlled modulation of oxygen availability (Hoffmann et al. 2021a). The availability of oxygen is a key process parameter in *B. subtilis* cultivations, strongly influencing cellular metabolism, redox balance, and energy generation (Nakano & Zuber 1998). From a bioprocess perspective, high oxygen demand during aerobic surfactin production represents a major challenge, as it increases foam formation through higher aeration and agitation, therefore making the operation much more challenging (Yao et al. 2015; Yeh et al. 2006). In particular, the loss of cultivation medium due to excessive foaming is highly likely (Théâtre et al. 2021). To avoid overfoaming, the usage of antifoaming agents or mechanical foam disruption is favoured (Davis et al. 2001; Hiller et al. 2025b). Especially the chemical antifoaming agents can increase the process costs, influence the cell physiology, inhibit the cell growth and lower the oxygen transfer rate which leads to even more aeration requirements (Koch et al. 1995; Yeh et al. 2006). From a process engineering perspective, the foaming issue associated with surfactin production has been addressed by anaerobic cultivation of *B. subtilis*, which allowed the complete omission of oxygen aeration (Hoffmann et al. 2020; Willenbacher et al. 2015). However, this approach resulted in high nitrite and acetate concentrations, which severely inhibited cell growth (Hoffmann et al. 2020).

Current bioprocesses for surfactin production are predominantly operated under aerobic conditions, as sufficient oxygen availability is required to sustain high growth rates and achieve elevated biomass concentrations (Ghiribi & Ellouze-Chaabouni 2011). Using sporulation-deficient *B. subtilis* strains in fed-batch bioreactor processes, surfactin titers of up to 26.5 g/L with a maximum product to biomass yield *Y*_P/X_ of 0.57 g/g were reported by Klausmann et al. (2021). Further optimization of aerobic fed-batch processes by variation of the feeding growth rate enabled titers of up to 36 g/L and *Y*_P/X_ values of 0.70 g/g (Hiller et al. 2024). Amin (2014) achieved with a wild-type strain in a fed-batch bioreactor process with exponential feeding 36 g/L crude biosurfactant titer and a *Y*_P/X_ of 1.13 g/g. A subsequent model-based process design approach further increased aerobic surfactin titers to 46.33 g/L while maintaining a *Y*_P/X_ of 0.69 g/g (Hiller et al. 2025b). Recent studies further demonstrated the potential of aerobic surfactin production, reporting titers of up to 52.6 g/L through metabolic reprogramming of central carbon metabolism and surfactin synthetase expression (Gao et al. 2026), and 32.5 g/L through multidimensional strain engineering, including fatty acid metabolism optimization and peptidoglycan modulation (Xia et al. 2025). In addition to process optimization, genetic approaches have been applied to improve surfactin production under aerobic conditions. Hermann et al. (2025) and Hiller et al. (2025a) established a two-phase process based on genetic code expansion, enabling an initial growth phase without surfactin formation followed by a combined growth and production phase. This strategy resulted in surfactin titers of 10.8 g/L and a *Y*_P/X_ of 0.13 g/g. Despite these high titers, aerobic processes typically require substantial substrate and energy investment for biomass formation, which is reflected in comparatively low product to biomass yields.

In contrast, anaerobic and oxygen-limited cultivation strategies have been shown to substantially increase *Y*_P/X_ values due to lower biomass formation. Willenbacher et al. (2015) reported a *Y*_P/X_ of 0.278 g/g under anaerobic conditions in bioreactor cultivations, while Hoffmann et al. (2021a) achieved *Y*_P/X_ values of up to 1.081 g/g in anaerobic shake flask experiments. Hoffmann et al. (2020) cultivated a *B. subtilis* strain in an anaerobic fed-batch bioreactor process, resulting in a *Y*_P/X_ of 0.15 g/g. However, these processes are generally associated with reduced growth rates, lower overall product titers, and physiological limitations, which restrict their applicability at larger scales. However, the systematic development and scale-up of such oxygen-switching strategies from screening-scale to bioreactor remains insufficiently explored, despite the fact that Hoffmann et al. (2021a) investigated several aerobic to anaerobic switching strategies at a bioreactor scale; these approaches resulted predominantly in the formation of non-productive biomass under anaerobic conditions.

Although aerobic and anaerobic surfactin production processes have been described previously, the targeted use of oxygen availability as a dynamic process control parameter remains insufficiently explored. In particular, it is still unclear whether an oxygen-responsive production strain can be used to shift surfactin biosynthesis from an aerobic biomass formation phase toward a defined oxygen-limited production phase. Such an approach could provide a process-oriented alternative to strictly anaerobic cultivation by maintaining limited oxygen availability while reducing the aeration intensity associated with fully aerobic surfactin production.

Oxygen availability was therefore investigated as a process control parameter to guide surfactin production in *B. subtilis*. For this purpose, a controlled aerobic to micro-aerobic switching strategy was developed and evaluated from screening-scale shake flask experiments to 30 L stirred-tank bioreactor conditions. The strategy is based on real-time monitoring of oxygen availability and employs the genetically engineered strain *B. subtilis* MG19, in which the native *srfA* promoter was replaced by the oxygen-responsive P_*nasD*_ promoter. This promoter exchange was designed to repress surfactin biosynthesis during aerobic biomass formation and to promote surfactin production under micro-aerobic conditions. In addition, transcriptional reporter strains carrying *lacZ* fusions to oxygen- and surfactin-associated promoters were used to characterise promoter activity in response to changing oxygen availability. The process concept was assessed using physiological, kinetic, and oxygen-based performance indicators and compared with a reference cultivation employing *B. subtilis* MG17. Together, this approach combines promoter engineering and oxygen-based process control to functionally decouple biomass formation and surfactin production under controlled bioreactor conditions.

## Materials and methods

### Chemicals and standards

Unless otherwise specified, all chemicals used in this study were obtained from Carl Roth GmbH & Co. KG (Karlsruhe, Germany). The surfactin standard (≥ 98% purity) was obtained from Sigma-Aldrich Laborchemikalien GmbH (Seelze, Germany).

### Bacterial strains

*B. subtilis* BMV9 (Vahidinasab et al. 2020) was the parental strain and based on this strain, four genetically modified derivative strains were constructed, each carrying defined reporter constructs to monitor the activity of selected promoters related to oxygen-limited and surfactin-associated metabolism. Strain *B. subtilis* MG15 harbours a *P*_*narG*_*-lacZ* transcriptional fusion integrated at the *amyE* locus and was used as a reporter for *narG* promoter activity. *B. subtilis* MG16 carries a *P*_*nasD*_*-lacZ* fusion at the amyE locus and served as a reporter for *nasD* promoter activity. *B. subtilis* MG17 contains a *P*_*srfA*_*-lacZ* fusion at the amyE locus and was applied to monitor the transcriptional activity of the surfactin synthetase operon. *B. subtilis* MG19 combines *P*_*nasD*_*-lacZ* reporter with a promoter exchange (*P*_*srfA*_*::P*_*nasD*_), enabling the analysis of surfactin gene expression under nitrate-responsive promoter control. All strains are derived from the sporulation-deficient background (*spo0A3*), are *sfp*^+^, and carry a deletion of *manPA*. A summary of the strains and their relevant genetic features is provided in Table 1.

**Table 1:** List of *Bacillus subtilis* strains with relevant characteristics and the important feature used in this study

Bacterial strains	Relevant characteristics	Feature	Reference
*B. subtilis* BMV9	*spo0A3; ΔmanPA; sfp*^+^	Initial strain	Vahidinasab et al. (2020)
*B. subtilis* MG15	BMV9; *amyE::[P*_*narG*_*-lacZ*, *spcR]*	Reporter strain for *P*_*narG*_	This study
*B. subtilis* MG16	BMV9; *amyE::[P*_*nasD*_*-lacZ*, *spcR]*	Reporter strain for *P*_*nasD*_	This study
*B. subtilis* MG17	BMV9; *amyE::[P*_*srfA*_*-lacZ*, *spcR]*	Reporter strain for *P*_*srfA*_	This study
*B. subtilis* MG19	MG16; *P*_*srfA*_*::P*_*nasD*_	Reporter strain for *P*_*nasD*_ and promoter exchange *P*_*srfA*_*::P*_*nasD*_	This study

### Shake flask cultivations

All shake flask cultivations were performed at 37 °C using an orbital incubator shaker (NewbrunswickTM/Innova 44, Eppendorf AG, Hamburg, Germany) operated at 180 rpm. Unless stated otherwise, cultivations were conducted in 1-L unbaffled Erlenmeyer flasks with varying filling volumes to modulate oxygen availability (Heyman et al. 2019; Schiefelbein et al. 2013). Pre-cultures were prepared uniformly for all experiments. Briefly, 10 mL of LB medium in 50-mL centrifuge tubes were inoculated with 50 µL of the respective cryostock and incubated overnight at 37 °C and 180 rpm. After approximately 13 h, the optical density at 600 nm (OD_600_) was determined. Main cultures were conducted in mineral salt medium (MSM) as described by Hoffmann et al. (2020), but in this study initially with 20 g/L of glucose and 0.1 M ammonia and nitrate concentrations. Cultures were inoculated from the overnight preculture to an initial OD_600_ of 0.1. Depending on the experimental setup, filling volumes of 5%, 10%, 25%, or 50% (v/v) were applied. Cultivations were carried out for up to 28 h, with samples taken at 4 h intervals unless stated otherwise. All cultivations were performed in biological triplicates. To measure the dissolved oxygen (pO_2_/DO) levels continuously via a single-use DO sensor pill and to monitor the real-time cell growth via the optical density using backscattered light at 940 nm (OD_940_), a multiparameter shaking system (MPS; DOTS Scientific Bioprocessing, INC., Wilkins Township, United States) was operated parallel. Shaking was performed at 180 rpm, corresponding to the optimal operating speed for the MPS. The factory-calibrated, single-use DO sensor pills are based on luminescence quenching and freely circulate within the cultivation medium, enabling continuous pO_2_ measurements over a range of 0–470% air saturation. During MPS cultivations, OD_940_ and pO_2_ were recorded every 20 s, with an acquisition time of 6 s for each optical density measurement. Signal fluctuations caused by shaking-induced movement of the medium and the sensor pill, as well as mechanical vibrations of the shaker platform, contributed to measurement noise and were considered during cultivation.

For comparative experiments among *B. subtilis* MG strains, MG15, MG16, and MG17 were cultivated using a filling volume of 50% (v/v) MSM. In experiments investigating the effect of oxygen availability, strains MG17 and MG19 were cultivated at filling volumes of 10%, 25%, and 50% (v/v). Fed-batch–like shake flask experiments were performed exclusively with strain MG19. Cultivations were initiated at a filling volume of 5% (v/v) and sequentially fed with MSM, not containing ammonia, until a final volume of 50% (v/v) was reached. For this purpose, the filling volume was initially increased to 10% (v/v), followed by 25% (v/v), and finally 50% (v/v), applied in intervals of 1 h, 2 h, and 3 h, respectively. Cultivations were performed using the multiparameter sensor system (MPS), with biomass formation (OD_940_) and dissolved oxygen (pO_2_/DO) monitored online. These measurements were used to evaluate which dissolved oxygen reduction profiles, induced by stepwise increases in filling volume, exerted a negative effect on cell growth. The best-performing profile was subsequently repeated in biological triplicates with sampling to enable the analysis of additional process parameters. Samples were collected after each feeding step and subsequently every 4 h.

The parental strain BMV9 was included in all experiments and treated identically to the respective test strains (Table 1). It served as a reference strain for Miller assay normalization.

### Bioreactor cultivations

For the bioreactor cultivations mainly the *B. subtilis* strain MG19 was used and for the reference process *B. subtilis* MG17. The preculture procedure was already described by Klausmann et al. (2021). Briefly, the first pre-culture was done in LB medium, while a chemically defined mineral salt medium was used for the subsequent second pre-culture. Shake flask cultivations were performed in an incubator shaker (NewbrunswickTM/Innova 44, Eppendorf AG, Hamburg, Germany) at 37 °C and 120 rpm. The second pre-culture was used to inoculate the bioreactor to an initial OD_600_ of 0.1.

Bioreactor cultures were carried out in a 30 L fermenter (ZETA GmbH, Graz/Lieboch, Austria). Two chemically defined mineral salt media were applied in the different cultivation runs and are hereafter referred to as M1 and M2. M1 corresponds to the mineral salt medium previously used for aerobic surfactin production by Klausmann et al. (2021), whereas M2 corresponds to the medium composition previously used for anaerobic surfactin production by Hoffmann et al. (2021a). In all bioreactor processes, glucose was used as the sole carbon source. Fed-batch cultivations were performed using a 50% (w/w) glucose feed solution. The bioreactor was operated at 37 °C and a controlled pH of 7.0, maintained with 4 M nitric or 4 M phosphoric acid and 20% (v/v) ammonia solution or 4 M sodium hydroxide as a base. The initial agitation speed was set to 300 rpm and aeration was supplied using sterile compressed air with values between 2 and 72 L/min. For cultivations performed with M1 (bioreactor runs A and B), an initial aeration rate of 2 L/min was applied, whereas cultivations with M2 (bioreactor runs C–G) were started with an aeration rate of 10 L/min. Based on the results obtained from the fed-batch-like shake flask experiments, a dynamic aerobic to micro-aerobic oxygen switching strategy was implemented in the bioreactor processes. Cultivations were initiated under aerobic conditions and subsequently transitioned to micro-aerobic conditions by stepwise reduction of the dissolved oxygen setpoint. The pO_2_ was decreased from an initial setpoint of either 50% air saturation (bioreactor runs C–G) or 20% air saturation (bioreactor runs A and B) to 20% at a specific optical density for 1 h (for aerobic medium), further to 10% after an additional hour, and finally to 1% air saturation to reach micro-aerobic conditions. The reduction in dissolved oxygen was achieved by automated reactor control through adjustment of agitation speed and aeration rate. Following completion of the batch phase, carbon feeding was initiated according to an exponential feeding strategy. The initial feed rate *F*_0_ and the feeding rate *F*(*t*) at every time point *t*, for the carbon feed was estimated directly after the batch phase according to the Eqs. (1) and (2) below. Here *F*_0_ is the initial feeding rate (kg/h); *F*(*t*) the feeding rate at every time point t (kg/h); *μ*_F_ the feeding growth rate (1/h); m the maintenance coefficient set to 0.05 g/(g*h); *Y*_X/S_ the substrate to biomass conversion yield in the batch phase (g/g); *C*_X,Batch_ the biomass concentration at the end of the batch phase (g/L); *V*_0_ the bioreactor filling volume at feed start (L); *C*_S,Feed_ the glucose concentration in the feed solution (g/L). An alternative feeding strategy, independent of the batch phase, involved initiating the feed directly at the beginning of the process without supplying glucose in the initial medium, following the approach described by Hiller et al. (2025b) (bioreactor runs *E* and *F*). Based on kinetic modelling, Hiller et al. (2025b) defined an initial feeding rate of 0.028 kg/h. This value was adopted in the present study and adjusted to 0.042 kg/h to account for the higher initial reactor filling volume. Subsequently, the exponential feeding profile described in Eq. (1) was applied.

1 $$F\left(t\right)= {F}_{0} *{e}^{{\mu}_{F} *t}$$

2 $${F}_{0}=\left(\frac{{\mu}_{F}}{{Y}_{X/S}}+m\right)* \frac{{C}_{X, Batch}* {V}_{0}}{{C}_{S,Feed}}$$

Table 2 summarizes the settings for the seven different bioreactor cultivations (A-G) of this study. The development of an aerobic to micro-aerobic switching process was scaled from shake flask experiments performed with the multiparameter sensor system (MPS) to stirred-tank bioreactor cultivations. For this purpose, *B. subtilis* MG19 was primarily used, as this strain enables suppression of surfactin production under aerobic conditions and a targeted shift of product formation to the micro-aerobic phase. Accordingly, all bioreactor experiments were conducted with strain MG19, except for bioreactor run G, in which the best-performing process configuration was repeated using strain MG17 as a reference for comparison. Bioreactor run A was performed using the M1 medium with an initial batch glucose concentration of 10 g/L. Once a dissolved oxygen level of 20% air saturation was reached, this setpoint was maintained for 1 h, followed by stepwise reductions to 10% for 1 h and finally to 1% air saturation to establish micro-aerobic conditions. Carbon feeding was initiated at the onset of the micro-aerobic phase using an exponential feeding strategy with a feeding growth rate of 0.1 1/h and a total volume of 7 L glucose solution. pH control was achieved using nitric acid and sodium hydroxide as titrants. Bioreactor run B followed the same operational strategy as run A; however, the feeding growth rate during the fed-batch phase was reduced to 0.05 1/h and the amount of feed solution increased to 10 L. Bioreactor run C was conducted using the M2 mineral salt medium with an initial batch glucose concentration of 25 g/L. Feeding was initiated immediately after depletion of batch glucose using a feeding growth rate of 0.3 1/h, while the dissolved oxygen concentration was maintained at 50% air saturation. Upon reaching an optical density of OD₆₀₀ = 100, the dissolved oxygen setpoint was reduced to 20% air saturation and held for 1 h. At this stage, the pH control system was switched from phosphoric acid and ammonia to nitric acid and sodium hydroxide. Subsequently, the dissolved oxygen concentration was further reduced to 10% air saturation for 1 h, followed by the addition of a sodium nitrate solution to achieve a final nitrate concentration of 0.1 M in the reactor. Thereafter, the dissolved oxygen level was reduced to 1% air saturation, the feed rate was decreased to 50% of the current value, and the feeding growth rate was lowered to 0.05 1/h. The initial batch volume was 20 L, and the final feed volume amounted to 10 L. Bioreactor run D was performed using the same operational strategy as run C, with the exception that the batch to feed volume ratio was adjusted to 1:1. In bioreactor runs E and F, batch glucose was omitted entirely, and feeding was initiated directly at the start of the cultivation (direct fed mode) using a feeding growth rate of 0.2 1/h. For run F, the feeding growth rate was reduced to 0.1 1/h upon reaching a dissolved oxygen concentration of 1% air saturation. Apart from these differences, the process configuration was identical to that applied in run D. Bioreactor run G was carried out using *B. subtilis* MG17 instead of MG19 as a reference strain. All other process parameters and operational strategies were identical to those applied in run D, but with a total feeding volume of only 10 L. Cultivations were terminated when the maximum working volume of the bioreactor was reached or when the predefined feed volume had been completely supplied.

**Table 2:** Summary of the settings and differences of the seven bioreactor cultivations of this study

Bioreactor cultivation	Strain	Mode	Media	Initial volume	Acid	Base	pO_2_ switch strategy	Feeding strategy	Addition of NaNO_3_
A	*B. subtilis* MG19	Fed-batch	M1	20 L	Nitric	Sodium hydroxide	20% → 10% → 1%	*F*_0_ via Eq. (2); *μ*_F_ = 0.1 1/h	No
B	*B. subtilis* MG19	Fed-batch	M1	20 L	Nitric	Sodium hydroxide	20% → 10% → 1%	*F*_0_ via Eq. (2); *μ*_F_ = 0.05 1/h	No
C	*B. subtilis* MG19	Fed-batch	M2	20 L	Phosphoric/nitric	Ammonia/sodium hydroxide	50% → 20% → 10% → 1%	*F*_0_ via Eq. (2); *μ*_F_ = 0.3 1/h → 0.05 1/h	Yes
D	*B. subtilis* MG19	Fed-batch	M2	15 L	Phosphoric/nitric	Ammonia/sodium hydroxide	50% → 20% → 10% → 1%	*F*_0_ via Eq. (2); *μ*_F_ = 0.3 1/h → 0.05 1/h	Yes
E	*B. subtilis* MG19	Direct fed mode	M2	15 L	Phosphoric/nitric	Ammonia/sodium hydroxide	50% → 20% → 10% → 1%	*F*_0_ = 0.042 kg/h; *μ*_F_ = 0.2 1/h	Yes
F	*B. subtilis* MG19	Direct fed mode	M2	15 L	Phosphoric/nitric	Ammonia/sodium hydroxide	50% → 20% → 10% → 1%	*F*_0_ = 0.042 kg/h; *μ*_F_ = 0.2 1/h → 0.1 1/h	Yes
G	*B. subtilis* MG17	Fed-batch	M2	15 L	Phosphoric/nitric	Ammonia/sodium hydroxide	50% → 20% → 10% → 1%	*F*_0_ via Eq. (2); *μ*_F_ = 0.3 1/h → 0.05 1/h	Yes

### Sample analysis

Samples taken during shake flasks or bioreactor cultivation were centrifuged at 3890 xg for 10 min at 4 °C (Multifuge X3R, Thermo Fisher Scientific, Waltham, USA). The cell-free supernatants were used to quantify glucose and ammonia with enzymatic assay kits (R-Biopharm AG, Darmstadt, Germany). Nitrate and nitrite were measured by ion chromatography at the Core Facility Hohenheim. The cell dry weight (CDW) was calculated from experimentally measured OD_600_ values with a correlation factor of 0.232 determined by Hiller et al. (2024) for the *B. subtilis* BMV9 strain. To measure the different promoter activities of the reporter strains (Table 1), a β-galactosidase assay was performed. The procedure and the formula to calculate the Miller units (MU) are described by Hoffmann et al. (2020).

### Surfactin quantification

The amount of surfactin formed by the strains during the shake flask and bioreactor cultivation processes was quantified by high-performance thin-layer chromatography (HPTLC) (CAMAG AG, Muttenz, Switzerland). All details are mentioned in Geissler et al. (2017). For the extraction, two mL of the centrifuged cell-free supernatant was added to a chloroform/methanol solution (2:1) in three procedure steps. The lower of the two phases was, after extraction, evaporated at 40 °C and 10 mbar using a rotary evaporator (RVC 2–25 Cdplus, Martin Christ Gefriertrocknungsanlagen GmbH, Osterode am Harz, Germany). The dried sample was dissolved in two mL of methanol and applied in 6-mm bands onto a silica HPTLC plate. Plate development was done over a migration distance of 60 mm using a mobile phase of chloroform/methanol/water (65:25:4). Surfactin detection was performed at 195 nm (Geissler et al. 2017), with a surfactin standard (Sigma Aldrich, Seelze, Germany) for quantification.

### Data analysis

The calculation of biological performance indicators was exclusively performed for the seven bioreactor runs (A–G) conducted as single determinations. Equations (3, 4, 5, 6, 7) describe the determination of product to biomass yield *Y*_P/X_ (g/g), product per substrate yield *Y*_P/S_ (g/g), biomass per substrate yield *Y*_X/S_ (g/g), specific productivity *q*_P/X_ (g/(g*h)), and space–time yield *P*_V_ (g/(h*L)) normalized to the working volume of the bioreactor. The calculations were based on the parameters biomass *X* (g), glucose as substrate *S* (g), product surfactin *P* (g), process time *t* (h), and the bioreactor working volume *V*_R_ (L). All biological performance indicators were calculated at the maximum values of biomass *X* and product *P* for shake flask cultivations, whereas for bioreactor cultivations they were determined at the endpoint of the respective process. Especially *Y*_P/X_ in shake flasks was calculated conservatively using *P*_max_ and *X*_max_.

3 $${Y}_{P/X}= \frac{\Delta P}{X}$$

4 $${Y}_{P/S}= \frac{\Delta P}{\Delta S}$$

5 $${Y}_{X/S}= \frac{\Delta X}{\Delta S}$$

6 $${q}_{P/X}= \frac{\Delta P}{X*t}$$

7 $${P}_{V}= \frac{\Delta P}{t* {V}_{R}}$$

To assess the efficiency of oxygen utilization for biomass formation at different filling volumes in shake flasks, oxygen-based conversion yields were determined for *B. subtilis* MG19. Specifically, the biomass to oxygen conversion yield *Y*_X/O2_ (g/g) was calculated at the respective maxima of biomass *X*_max_ (g). The amount of oxygen consumed ΔO_2_ (g) was estimated based on the measured dissolved oxygen signal pO_2,i_ (%), which was recorded using the MPS. First, the dissolved oxygen concentration *C*_O2,i_ (g/L) at the starting point of the cultivation (*t* = 0 h) and at the time point corresponding to *X*_max_ was calculated using Eq. (8). The volumetric oxygen transfer coefficient *k*_L_a_i_ (1/s), defined as the product of the liquid side mass transfer coefficient *k*_L_ and the specific gas–liquid interfacial area a, describes the capacity of the system to transfer oxygen from the gas into the liquid phase. Starting from a reference *k*_L_a (0.02 1/s) value determined at a filling volume of 10%, together with the corresponding filling volume (*V* = 0.1 L) and liquid diameter at maximum filling height (*d* = 12 cm), the *k*_La_ values for filling volumes of 25% and 50% were calculated using Eq. (9). For these calculations, filling volumes *V*_i_ of 0.25 L and 0.5 L and liquid diameters *d*_i_ of 11.5 cm and 10 cm were applied for 25% and 50%. Subsequently, the oxygen transfer rate OTR_i_ (g/(L*s)) was calculated using Eq. (10), where *C*_O2_^*^ (0.0067 g/L) represents the oxygen saturation concentration in the liquid phase. Based on the calculated OTR values, the oxygen uptake rate OUR_i_ (g/s) was determined using Eq. (11), assuming a constant rate of change in dissolved oxygen concentration Δ*C*_O2_/Δ*t* (g/(L*s)). Finally, the total amount of oxygen consumed ΔO_2_ (g) was calculated using Eq. (12), representing the cumulative oxygen uptake required for the formation of the respective maximum amount of biomass. Equation (13) describes the corresponding oxygen consumption per unit of biomass.

8 $${C}_{{O}_{2}, i}= \frac{{pO}_{2, i}}{100\%}* {{C}_{{O}_{2}}}^{*}$$

9 $${k}_{L}{a}_{i}=0.02 \frac{1}{s}* \frac{0.1 L}{{V}_{i}}* \frac{{d}_{i}^{2}}{12 cm}$$

10 $${OTR}_{i}= {k}_{L}{a}_{i}*({C}_{{O}_{2}}^{*}- {C}_{{O}_{2,i}}$$

11 $${OUR}_{i}= {OTR}_{i}- \frac{\Delta {C}_{{O}_{2}}}{\Delta t}$$

12 $$\Delta {O}_{2}= \frac{{OUR}_{i}\left(t=o h\right)+ {OUR}_{i} (t\left({X}_{max}\right))}{2}* \Delta t$$

13 $${Y}_{X/{O}_{2}}= \frac{\Delta X}{\Delta {O}_{2}}$$

### Plotting of experimental data

All graphs were generated using the OriginPro 2022b software (OriginLab Corporation, Northampton, USA).

## Results

### Comparison of promoter activity

**Fig. 1:**
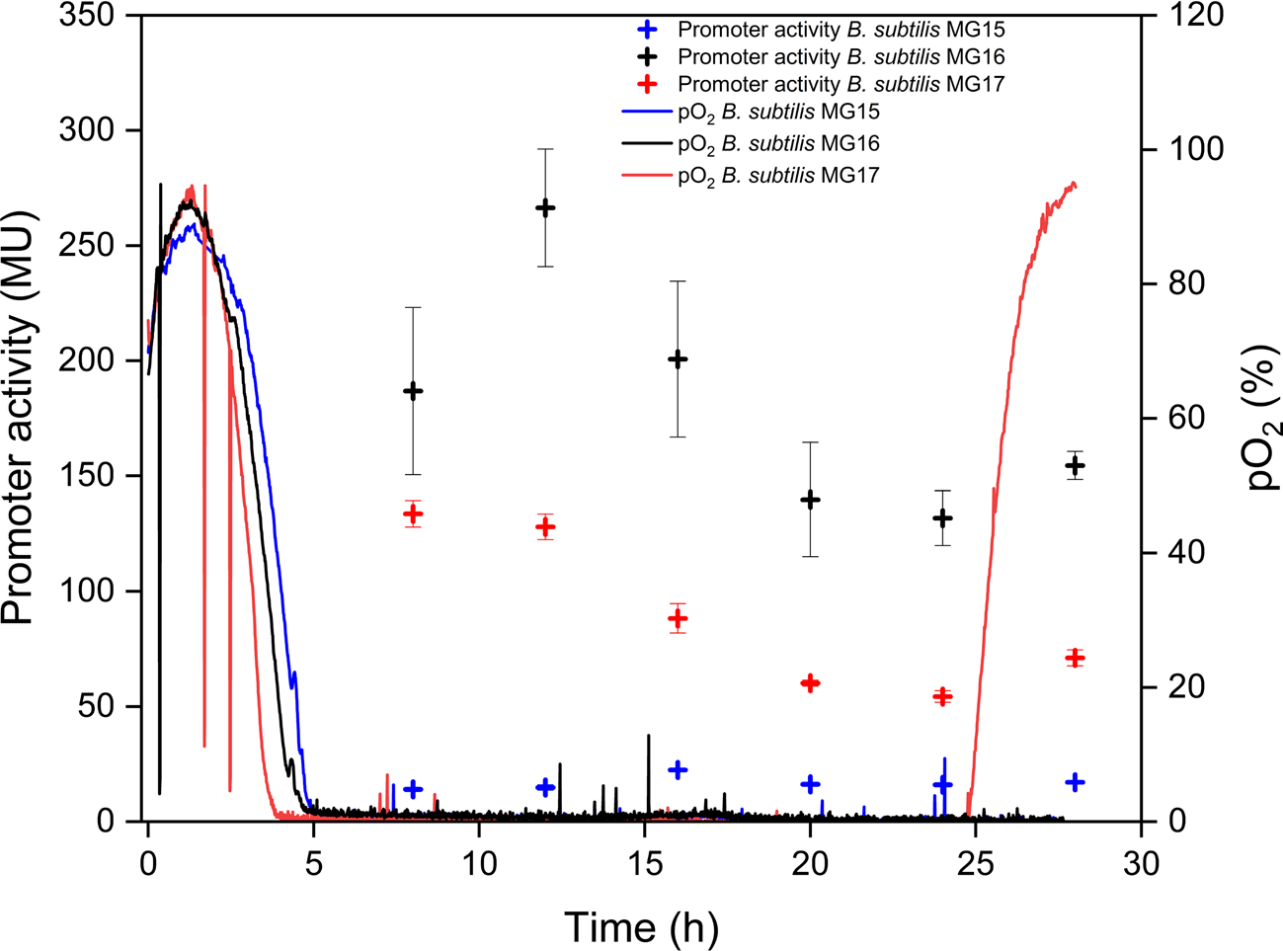
Comparison of dissolved oxygen profiles (pO_2_) and promoter activities of *Bacillus subtilis* reporter strains in shake flask cultivations with 50% (v/v) filling volume. Online pO_2_ signals (%) were recorded using the multiparameter sensor system (MPS) for *B. subtilis* MG15 (*P*_*narG*_*–lacZ*, blue line), *B. subtilis* MG16 (*P*_*nasD*_*–lacZ*, black line), and *B. subtilis* MG17 (*P*_*srfA*_*–lacZ*, red line). Promoter activities were quantified by Miller assay and are shown as Miller units (MU) at selected sampling times for *B. subtilis* MG15 (blue crosses), *B. subtilis* MG16 (black crosses), and *B. subtilis* MG17 (red crosses)

To compare the oxygen-dependent promoter regulation, the reporter strains (Table 1) *B. subtilis* MG15, MG16, and MG17 were cultivated in shake flasks while monitoring the dissolved oxygen profile (pO_2_) online using the MPS. In all cultivations, the dissolved oxygen decreased rapidly from initially high values around 90% to near zero within the first 4–5 h, indicating the establishment of oxygen-limited (micro-aerobic) conditions. After reaching nearly 0% pO_2_ levels, the dissolved oxygen remained close to zero for MG15 and MG16 throughout the cultivation, whereas *B. subtilis* MG17 exhibited a pronounced increase of pO_2_ after 25 h, suggesting strongly reduced oxygen uptake in the late phase of cultivation (Fig. 1). Promoter activities quantified by Miller assay revealed clear differences between the tested promoters under oxygen limitation (Fig. 1). The promoter *P*_*nasD*_ from *B. subtilis* MG16 showed the highest activity and remained strongly induced during the oxygen-limited phase, reaching the maximum at 12 h with around 260 MU and subsequently decreasing only moderately over time. In contrast, the promoter *P*_*narG*_ in *B. subtilis* MG15 displayed only low activity throughout the cultivation, with Miller units remaining close to baseline compared to MG16. The native surfactin promoter *P*_*srfA*_ in *B. subtilis* MG17 exhibited intermediate activity levels during the oxygen-limited phase and gradual decrease over time, indicating a less pronounced oxygen-dependent induction than observed for *P*_*nasD*_, with a maximum after 8 h at around 140 MU and therefore nearly half of the activity compared to *P*_*nasD*_. Overall, these results confirm *P*_*nasD*_ as the most responsive and strongest promoter under oxygen limitation or micro-aerobic conditions among the promoters tested, whereas *P*_*narG*_ showed only minor induction under applied conditions.

### Oxygen-dependent performance of *B. subtilis* MG17 and MG19 in shake flask cultivations

**Fig. 2:**
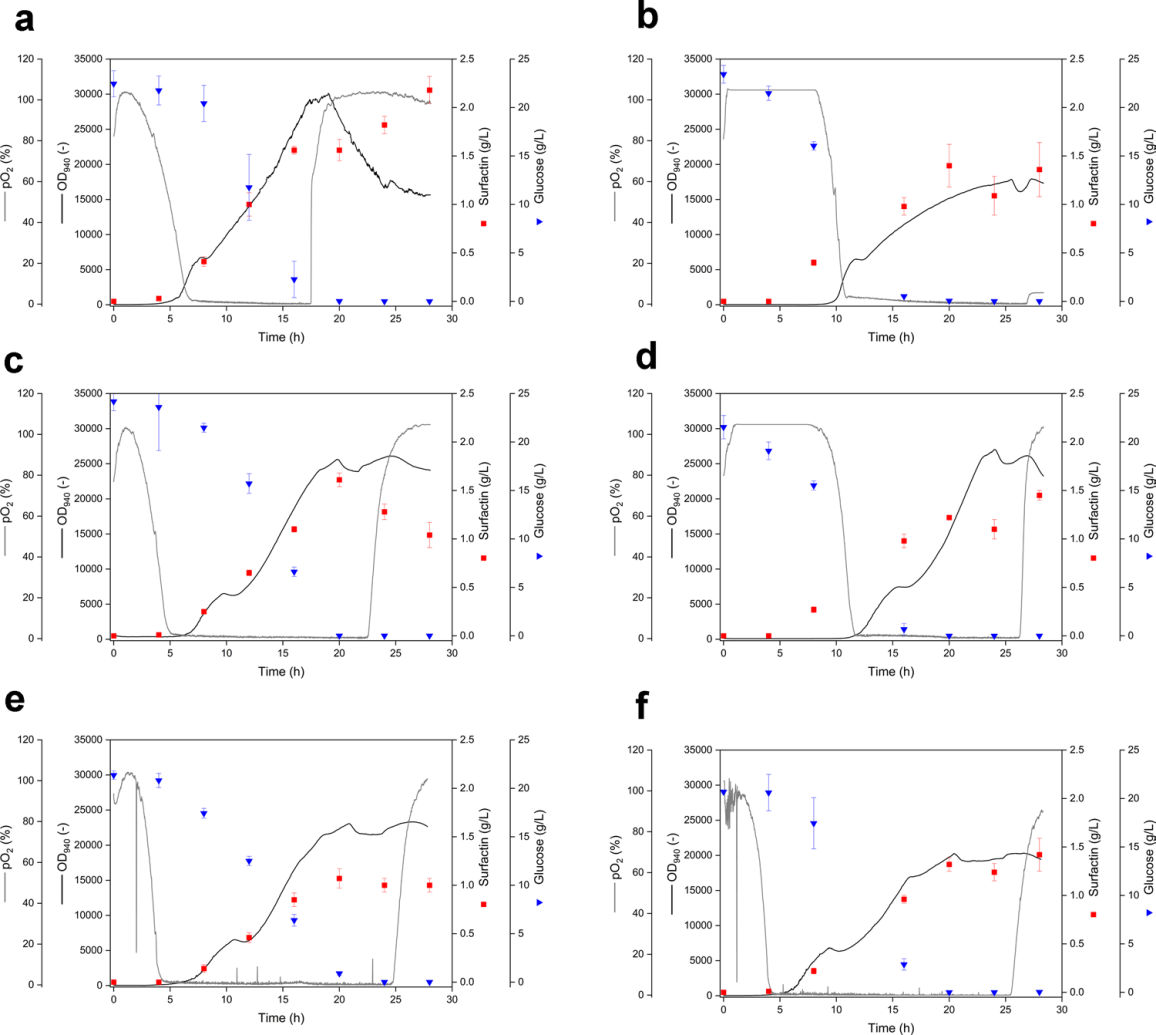
Comparison of *Bacillus subtilis* MG17 and MG19 shake flask cultivations under different oxygen availabilities (filling volumes). Online profiles of dissolved oxygen pO_2_ (grey line) and biomass signal backscatter-based OD_940_ (black line) were recorded using the multiparameter sensor system (MPS). Surfactin concentrations (red squares) and glucose concentrations (blue inverted triangles) were determined from sampled culture broth at the indicated time points. **A** MG17, 10% (v/v); **B** MG19, 10% (v/v); **C** MG17, 25% (v/v); **D** MG19, 25% (v/v); **E** MG17, 50% (v/v); **F** MG19, 50% (v/v)

Shake flask cultivations of *B. subtilis* MG17 and MG19 were performed at filling volumes of 10%, 25%, and 50% (v/v) to impose different oxygen availabilities, while pO_2_ and biomass formation (OD_940_) were monitored online using the MPS (Fig. 2 A–F). Across all filling volumes, pO_2_ decreased to oxygen-limited levels within five to ten hours for both strains, while glucose consumption coincided with increasing OD_940_ signals, confirming growth-associated substrate utilization (Fig. 2A–F). Late-phase increases in pO_2_ were observed in several cultivations and were consistent with reduced oxygen uptake after substrate depletion or entry into stationary phase. Surfactin accumulation occurred predominantly during the oxygen-limited phases. At 10% (v/v), MG17 reached the highest surfactin concentrations among the tested conditions, whereas MG19 produced lower surfactin levels under the highest oxygen availability (Fig. 2A and B), consistent with oxygen-dependent suppression of surfactin formation in MG19. At 25% (v/v), both strains produced comparable final surfactin concentrations (Fig. 2C and D), while at 50% (v/v), surfactin concentrations were overall reduced and approached a plateau earlier (Fig. 2E and F).

The calculated biological performance indicators quantified these differences (Table 3). Biomass to substrate yields *Y*_X/S_ varied only moderately across all experiments (0.13–0.19 g/g), indicating comparable overall conversion of glucose into biomass. In contrast, product to biomass yields *Y*_P/X_ decreased with increasing filling volume for both strains, with MG17 showing the highest *Y*_P/X_ at 10% (v/v) (0.61 g/g) and lower values at 25% and 50% (v/v) (0.47 and 0.32 g/g, respectively). MG19 displayed lower *Y*_P/X_ values at 10% and 25% (v/v) (0.46 g/g in both cases) and a reduced value at 50% (v/v) (0.35 g/g). Substrate-based product yields *Y*_P/S_ ranged from 0.05 to 0.10 g/g, with the highest value observed for MG17 at 10% (v/v) (0.10 g/g). Oxygen-based biomass yields increased strongly with decreasing oxygen availability, reflected by *Y*_X/O2_ increasing from 0.76 to 3.87 g/g for MG17 and from 0.64 to 4.63 g/g for MG19 when comparing 10 to 50% (v/v), consistent with progressively reduced oxygen supply. Specific productivities *q*_P/X_ were highest for MG17 at 25% (v/v) (0.024 g/(g*h)), whereas MG19 showed comparable values at 10% and 25% (v/v) (0.016 g/(g*h)) and a lower value at 50% (v/v) (0.013 g/(g*h)).

**Table 3:** Biological performance indicators for *Bacillus subtilis* MG17 and MG19 under different oxygen availabilities (filling volumes) in shake flask cultivations

Strain and condition	*Y*_X/S_ (g/g)	*Y*_P/X_ (g/g)	*Y*_P/S_ (g/g)	*Y*_X/O2_ (g/g)	*q*_P/X_ (g/g*h)
*B. subtilis* MG17 10% (v/v)	0.18	0.61	0.10	0.76	0.022
*B. subtilis* MG19 10% (v/v)	0.13	0.46	0.06	0.64	0.016
*B. subtilis* MG17 25% (v/v)	0.14	0.47	0.07	1.52	0.024
*B. subtilis* MG19 25% (v/v)	0.15	0.46	0.07	1.90	0.016
*B. subtilis* MG17 50% (v/v)	0.16	0.32	0.05	3.87	0.016
*B. subtilis* MG19 50% (v/v)	0.19	0.35	0.07	4.63	0.013

### Evaluation of DO reduction profiles for fed batch–like shake flasks cultivations of B. subtilis MG19

**Fig. 3:**
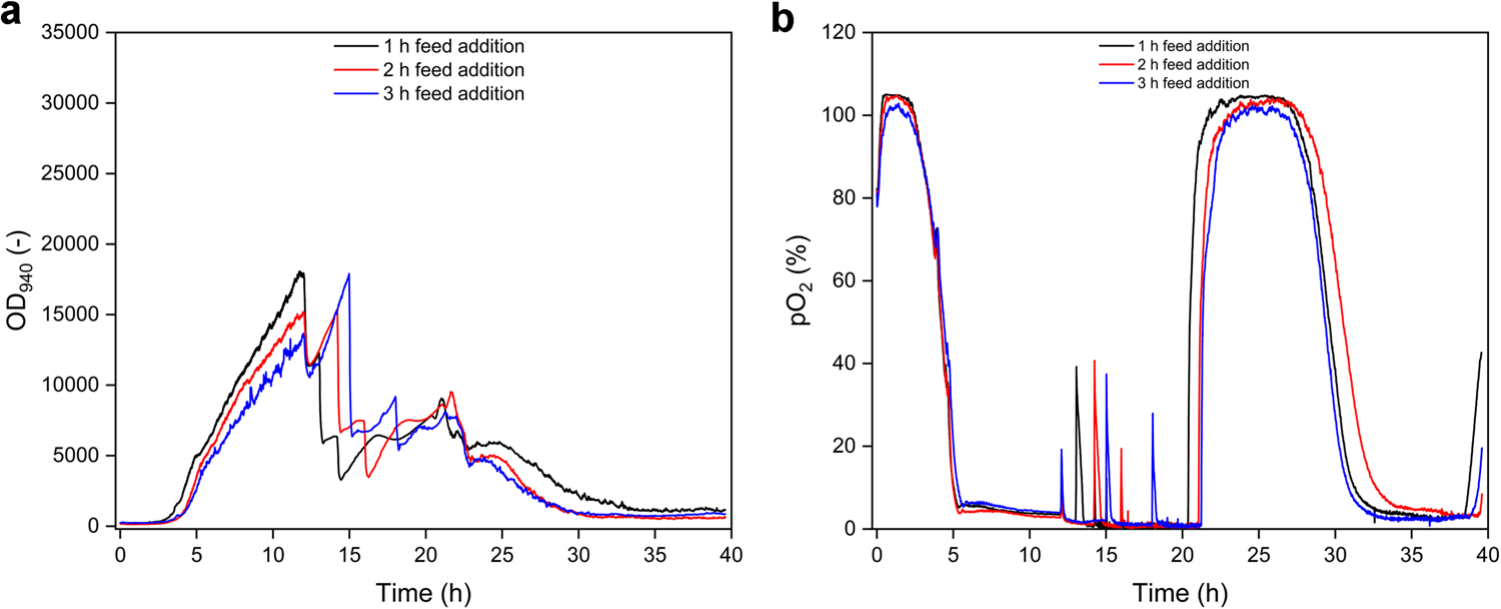
Screening of dissolved oxygen reduction profiles in fed-batch–like shake flasks cultivations of *Bacillus subtilis* MG19 using stepwise increases in filling volume. Cultivations were initiated at 5% (v/v) and the filling volume was increased stepwise to 10%, 25%, and 50% (v/v) using three different feeding intervals: 1 h (black), 2 h (red), and 3 h (blue). **A** Online biomass signal recorded as backscatter-based OD_940_. **B** Online dissolved oxygen profile (pO_2_) measured with the MPS. Data represent screening experiments without sampling

To evaluate whether the rate of dissolved oxygen (DO) reduction affects growth stability during the transition from aerobic to micro-aerobic conditions, fed-batch–like shake flask cultivations with *B. subtilis* MG19 were conducted using stepwise increases in filling volume. Three different feeding intervals (1 h, 2 h, and 3 h) were compared, and biomass formation (OD_940_) as well as pO_2_ were monitored online using the MPS (Fig. 3A and B). Across all three profiles, highly similar cultivation patterns were observed. In each experiment, pO_2_ decreased from initially high values to near-zero levels within the first hours of cultivation and remained oxygen-limited during the subsequent phases (Fig. 3B). Stepwise increases in filling volume induced only minor transient fluctuations in the pO_2_ signal, but the overall DO trajectories remained comparable between the tested feeding intervals. Likewise, OD_940_ profiles exhibited similar growth behaviour, with a continuous increase during the growth phase and no indication of growth collapse or abrupt signal loss at any time point (Fig. 3A). Overall, the screening experiments indicated that the tested DO reduction rates did not cause detectable differences in biomass formation or signs of cell death. Thus, within the investigated range, *B. subtilis* MG19 tolerated the applied oxygen reduction profiles, suggesting that the exact timing of the stepwise volume increase was not a critical factor for growth stability under the applied conditions.

**Fig. 4:**
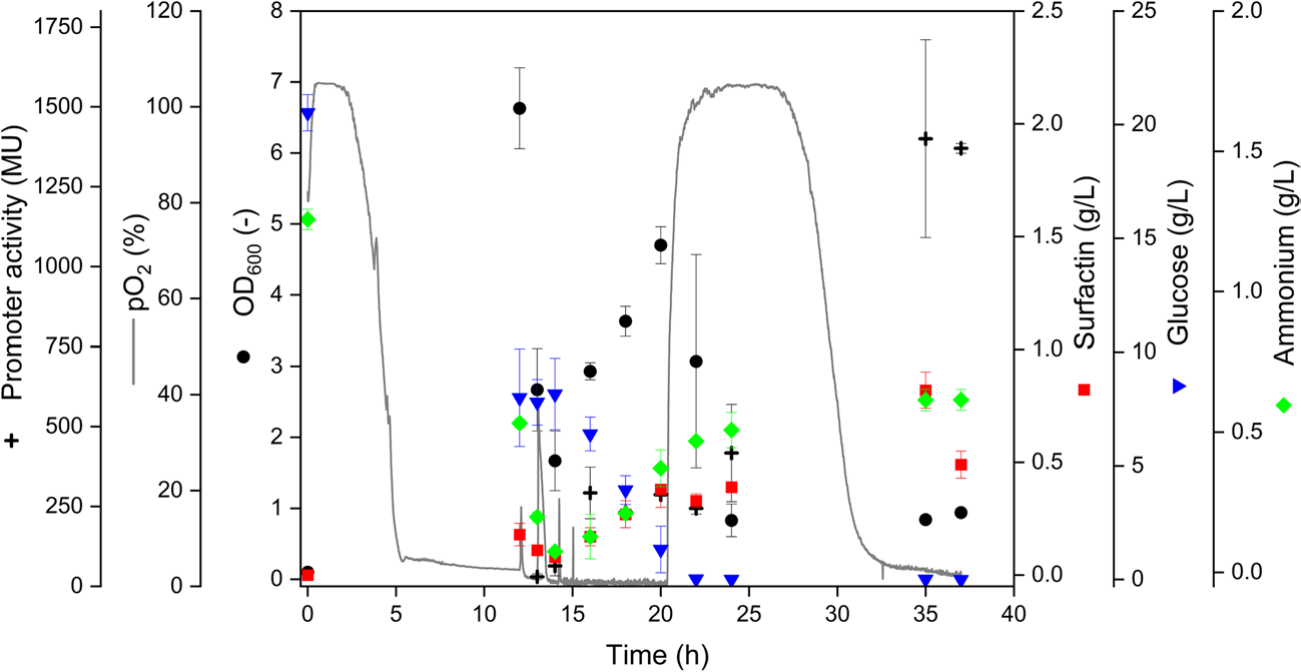
Fed-batch–like shake flask cultivation of *Bacillus subtilis* MG19 using the one hour feeding interval with sampling. The cultivation was initiated at 5% (v/v) and the filling volume was increased stepwise to 10%, 25%, and 50% (v/v) at one hour intervals by sequential feed additions. The dissolved oxygen profile (pO_2_, grey line) was recorded online using the MPS. Biomass was determined offline as OD_600_ (black circles). Promoter activity was quantified by Miller assay and is shown as Miller units (black crosses). Surfactin concentration (red squares), glucose concentration (blue inverted triangles), and ammonium concentration (green diamonds) were determined from sampled culture broth at the indicated time points

In order to complement the online screening experiments, the fed-batch–like shake flask cultivation using the 1 h feeding interval was repeated in biological triplicates with sampling to quantify biomass formation, substrate consumption, surfactin production, and promoter activity (Fig. 4). The pO_2_ profile showed an initially aerobic phase followed by a rapid decrease to oxygen-limited conditions within the first hours of cultivation. After the stepwise volume increase and feeding phase, pO_2_ remained close to zero for an extended period, before returning to near air saturation in the late cultivation phase, consistent with a reduced oxygen uptake after substrate depletion and entry into stationary phase. Offline measurements confirmed growth-associated substrate consumption. OD_600_ increased during the oxygen-limited phase and reached its maximum during the mid-cultivation period, while glucose concentrations continuously decreased and approached depletion toward the late cultivation phase (Fig. 4). Surfactin accumulation started during the oxygen-limited phase and increased further over time, reaching the highest concentration of around 1 g/L at the end of the cultivation. Ammonium concentrations decreased during the main growth phase and increased again toward the end of the cultivation, indicating changing nitrogen availability over time (Fig. 4). Promoter activity quantified by Miller assay remained low during the initial cultivation phase and increased markedly under oxygen-limited conditions, reaching the highest value of around 300 MU at the end of the process. Overall, the sampled cultivation confirmed that the applied stepwise volume increase with 1 h intervals enabled a reproducible transition into oxygen limitation and supported surfactin formation under the intended oxygen regime without dying cells.

### Scale-up of the aerobic to micro-aerobic switching strategy to 30-L bioreactor cultivations

**Fig. 5:**
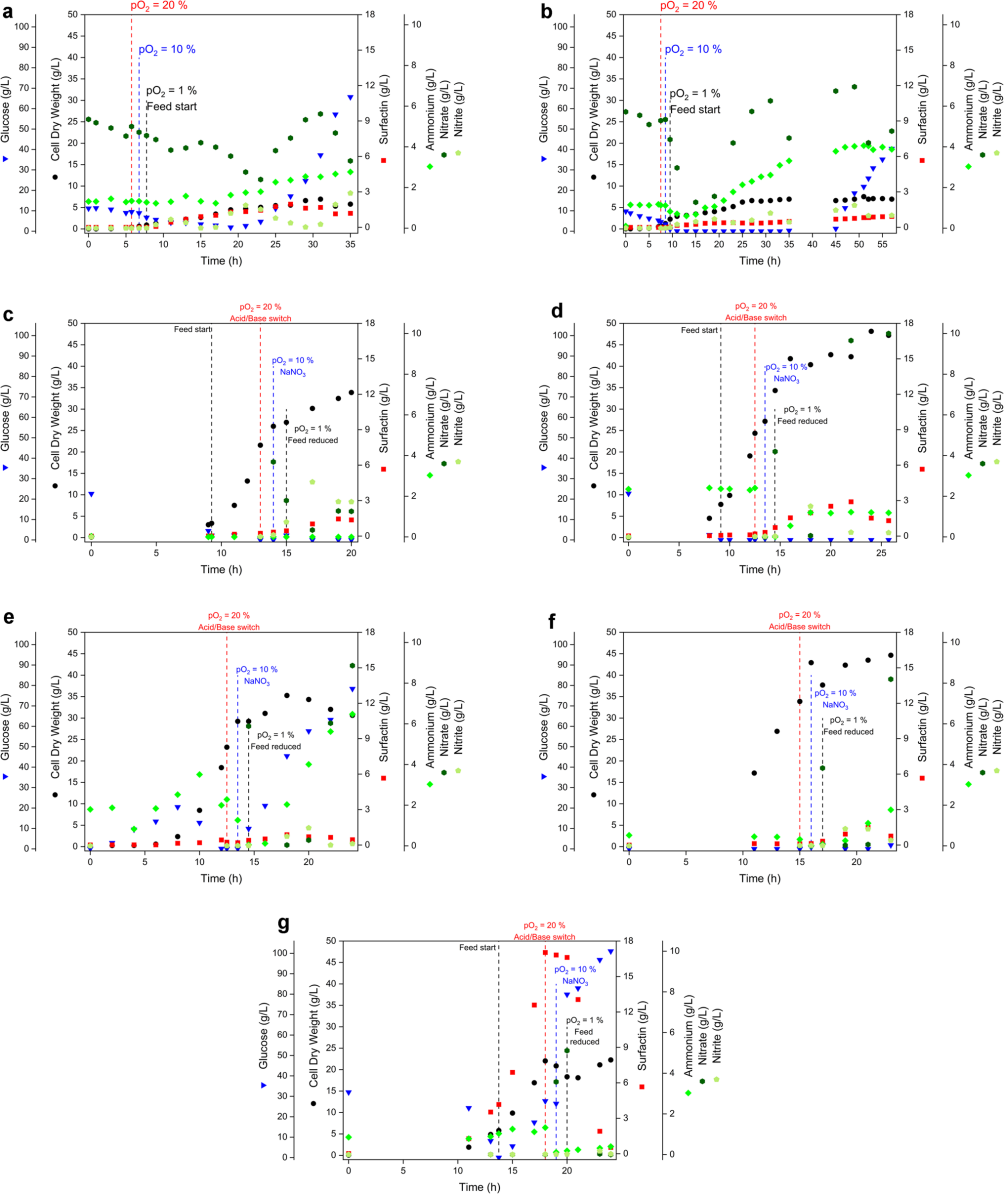
Overview of the seven bioreactor cultivations (**A**–**G**) performed in this study. Details of the experimental conditions are provided in Table 2 of this study. Runs A–F were performed with *Bacillus subtilis* MG19, and run G with *Bacillus subtilis* MG17. Shown are cell dry weight (black circles), surfactin concentration (red squares), glucose concentration (blue inverted triangles), ammonium concentration (green diamonds), nitrate concentration (dark green hexagons), and nitrite concentration (light green pentagons). Key process switches, including changes in dissolved oxygen setpoints, nitrate addition, and feed-related adjustments, are indicated directly in the individual panels*.* All experiments were conducted as single determination

Following the shake flask screening, the aerobic to micro-aerobic switching concept was implemented at bioreactor scale in a series of seven 30 L cultivations (runs A–G). The runs were designed to compare the influence of medium type, cultivation strategy, namely fed-batch and direct-fed mode (omission of batch phase) processes, and feeding concept on growth and surfactin formation under controlled conditions. *B. subtilis* MG19 was used for runs A–F to shift surfactin formation toward the micro-aerobic phase, whereas run G was performed with *B. subtilis* MG17 as a reference under the best-performing process configuration. An overview of the experimental setups is provided in Fig. 5, with full process settings summarised in Table 2. Run A (Fig. 5A) was carried out with *B. subtilis* MG19 in M1 mineral salt medium. During the initial batch phase, glucose decreased from approximately 10 g/L to near-zero within the first hours of cultivation, while cell dry weight increased to around 2 g/L. After the transition to micro-aerobic conditions at a pO₂ setpoint of 1% at around 8 h, biomass formation continued and cell dry weight increased to approximately 5–6 g/L by 20–25 h, reaching about 7 g/L in the later cultivation phase. Surfactin accumulation remained low throughout the process and increased only gradually, approaching values below 1 g/L toward the end of cultivation. Nitrate was present at several g/L and decreased during the cultivation, whereas ammonium increased progressively to approximately 3–4 g/L; nitrite remained low and only showed minor accumulation. In the late process phase, glucose accumulated strongly and reached values above 60 g/L, indicating limited substrate uptake under the applied conditions. Run B (Fig. 5B) was performed with *B. subtilis* MG19 in M1 mineral salt medium using the aerobic to micro-aerobic switching strategy and exponential feeding. During the initial batch phase, glucose decreased from approximately 10 g/L to near zero within the first ~ 10 h, while cell dry weight increased to around 3 g/L. After establishment of micro-aerobic conditions at a pO_2_ setpoint of 1% after 10 h, the fed-batch phase was initiated. In comparison to run A, the feeding growth rate was reduced to 0.05 1/h in order to mitigate glucose accumulation in the micro-aerobic phase. Following the switch, biomass increased further and approached a plateau at approximately 6–7 g/L between 25 and 35 h, remaining largely constant thereafter. Surfactin formation stayed low throughout the process and increased only gradually, reaching below 1 g/L at the endpoint. Nitrate was initially present at roughly 5–6 g/L, stayed stable under aerobic conditions and declined to around 2 g/L after the onset of micro-aerobic conditions, whereas ammonium increased continuously to approximately 4 g/L; nitrite remained below 1 g/L over the entire cultivation. In the late phase after approximately 45 h, glucose accumulated markedly up to around 30–35 g/L, indicating reduced substrate uptake under the applied conditions despite the reduced feeding growth rate. After the initial aerobic growth phase in M2 mineral salt medium, run C (Fig. 5C) aimed to shift *B. subtilis* MG19 from biomass formation toward micro-aerobic, nitrate-supported production conditions. Biomass increased rapidly, with cell dry weight rising to approximately 25–27 g/L by around 14–15 h and further to about 33–34 g/L by 20 h. In parallel, glucose was efficiently consumed and decreased from the initial level to values close to zero. At approximately 15 h, the cultivation was switched to micro-aerobic operation at a pO_2_ setpoint of 1%. This transition was accompanied by a reduction of the feeding growth rate from the initial high-growth setting of 0.3 1/h to a lower value of 0.05 1/h and a concomitant decrease of the ongoing feed rate to half of the current value to avoid substrate accumulation. To promote nitrate respiration and trigger product formation under oxygen limitation, NaNO_3_ was added at the onset of the micro-aerobic phase and acid/base were changed. Following this addition, nitrate became detectable and subsequently declined, while nitrite transiently accumulated, indicating active nitrate reduction; ammonium remained close to zero over the depicted time course. Surfactin concentrations increased gradually during the micro-aerobic phase and reached approximately 1–2 g/L by 20 h, indicating only moderate product formation under the applied switching and feeding conditions. Building on the process configuration of run C, run D (Fig. 5D) applied the same mineral salt medium, oxygen switching strategy, and nitrate supplementation, but with an increased overall feed volume. Biomass formation was strongly enhanced and cell dry weight increased steeply during the aerobic phase, reaching approximately 34 g/L at the time point when the process was shifted to micro-aerobic operation at a pO_2_ setpoint of 1% around 15 h. After the switch, biomass continued to increase for 1 h but stayed stable afterward at values of approximately 40–45 g/L during the subsequent cultivation period. Glucose was rapidly consumed and remained close to zero throughout the time course after the onset of feeding, indicating carbon-limited operation without glucose accumulation. At the onset of the micro-aerobic phase, NaNO_3_ addition resulted in a sharp increase of nitrate, followed by a rapid decline, while nitrite showed a transient accumulation at later time points, consistent with nitrate reduction activity. Ammonium concentrations decreased after the switch and remained at comparatively low levels during the later cultivation phase. Surfactin formation was initiated during the micro-aerobic phase and increased to approximately 3 g/L, reaching its maximum around 23 h, followed by a decrease toward the end of the cultivation period shown. In run E (Fig. 5E), the process was initiated without a batch glucose phase and carbon supply was started immediately at the beginning of the cultivation using exponential feeding with a constant feeding growth rate of 0.2 1/h. Biomass formation increased rapidly during the first half of the cultivation, with cell dry weight rising steeply from the first sampling point to approximately 30 g/L by around 15 h, followed by a further increase to roughly 35 g/L at later time points. The transition to micro-aerobic operation at a pO_2_ setpoint of 1% occurred at 15 h and coincided with the onset of nitrate supplementation to promote nitrate respiration under oxygen limitation. After nitrate addition, nitrate became detectable in the broth and subsequently changed over time, while nitrite remained low and only showed minor accumulation. Ammonium concentrations increased during the cultivation and reached values in the range of several g/L toward the end of the process. Glucose concentrations were low in the early phase but increased markedly after the switch toward micro-aerobic conditions, reaching high values of 80 g/L at the final sampling points, indicating that glucose feeding exceeded the instantaneous uptake capacity under the applied conditions. Surfactin concentrations increased only slightly over time and remained below 1 g/L throughout the cultivation period shown. Run F (Fig. 5F) followed the same process concept as run E, with carbon feeding initiated at the start of the cultivation in the absence of a batch glucose phase. Biomass formation increased strongly over time and cell dry weight reached approximately 45 g/L by around 17 h, and stayed stable afterward. The process was shifted to micro-aerobic operation at a pO_2_ setpoint of 1% at 17 h, and NaNO₃ was added at the onset of this phase to promote nitrate respiration. In contrast to run E, the feeding growth rate was reduced from 0.2 1/h upon reaching pO_2_ = 1% in order to avoid the pronounced glucose accumulation previously observed. Consistent with this adjustment, glucose remained close to zero throughout the cultivation, indicating carbon-limited operation without substrate accumulation. After nitrate addition, nitrate became detectable and increased markedly, while nitrite remained low and showed only minor accumulation; ammonium increased to moderate levels during the later cultivation phase. Surfactin formation remained low and increased only slightly over time, staying below 1 g/L throughout the depicted process. In run G (Fig. 5G), the process configuration applied in run D was repeated, but *B. subtilis* MG17 was used as reference strain instead of *B. subtilis* MG19. In contrast to the *B. subtilis* MG19 based runs, surfactin formation was already detectable during the aerobic phase and increased strongly prior to the transition toward micro-aerobic operation, reaching high concentrations of up to 17 g/L before the pO_2_ setpoint was lowered to 1% at 20 h. After the reduction profile of the dissolved oxygen and the switch to micro-aerobic conditions, a pronounced process disturbance became apparent. Glucose concentrations increased rapidly and accumulated to high levels of up to 100 g/L, indicating that substrate uptake was strongly impaired under the applied micro-aerobic conditions. At the same time, surfactin concentrations decreased sharply, consistent with rapid loss of product after the transition. Despite these changes in carbon balance and product concentration, cell dry weight remained comparatively stable and did not show a corresponding collapse over the time period shown. Nitrate became detectable around the transition and nitrite remained low, while ammonium stayed at low concentrations. Overall, the reference strain *B. subtilis* MG17 did not tolerate the applied switch toward micro-aerobic conditions in the same manner as *B. subtilis* MG19, resulting in glucose accumulation and an abrupt decline of surfactin after the transition, while biomass levels remained largely unaffected.

Table 4 summarises endpoint-based biological performance indicators for all bioreactor cultivations, calculated from biomass (*X*), glucose (*S*), and surfactin (*P*) at the end of each process. Across the MG19 runs (A–F), surfactin endpoint concentrations *P*_max_ ranged from 0.88 to 2.91 g/L, with the highest *P*_max_ obtained in run D. The reference run with MG17 (G) reached a substantially higher *P*_max_ of 17.03 g/L, but in the aerobic phase. Biomass to substrate yields *Y*_X/S_ varied between 0.06 and 0.23 g/g for MG19 and were 0.20 g/g for MG17. Product-related yields *Y*_P/X_ and *Y*_P/S_ were low for most bioreactor runs, with *Y*_P/X_ ranging from 0.01 to 0.21 g/g and *Y*_P/S_ from 0.003 to 0.02 g/g, respectively. Space–time yield *P*_V_ ranged from 0.016 to 0.051 g/(L*h), with the highest value observed in run D (0.051 g/(L*h)); the MG17 reference run showed a *P*_V_ of 0.022 g/(L*h). The cellular productivity *q*_P/X_ was in the range of 0.0004 to 0.0023 g/(g*h).

**Table 4:** Biological performance indicators for *Bacillus subtilis* MG19 (A–F) and MG17 (G) in different bioreactor cultivations

Bioreactor run	*P*_max_ (g/L)	*Y*_X/S_ (g/g)	*Y*_P/X_ (g/g)	*Y*_P/S_ (g/g)	*P*_V_ (g/(L*h))	*q*_P/X_ (g/g*h)
A	1.96	0.09	0.21	0.02	0.034	0.006
B	0.92	0.06	0.13	0.008	0.016	0.0023
C	1.45	0.21	0.04	0.008	0.041	0.002
D	2.91	0.23	0.03	0.006	0.051	0.0012
E	0.88	0.22	0.01	0.003	0.019	0.0004
F	1.49	0.21	0.02	0.004	0.022	0.0009
G	17.03	0.20	0.02	0.005	0.022	0.0008

Details to the experimental setup is given in Table 2 of this study

## Discussion

Oxygen availability constitutes a central control variable in *B. subtilis* processes because it directly shapes respiratory activity and the regulatory response associated with nitrate respiration (Härtig & Jahn 2012; Nakano et al. 1998). The first part of this study therefore focused on promoter behaviour under oxygen-limited conditions in shake flasks, providing the regulatory basis for an oxygen-responsive production phenotype and subsequent transfer to bioreactor operation. Promoter activity analyses using the reporter strains *B. subtilis* MG15 (*P*_*narG*_*–lacZ*), MG16 (*P*_*nasD*_*–lacZ*), and MG17 (*P*_*srfA*_*–lacZ*) demonstrated that oxygen limitation differentially affects respiratory and surfactin-related expression patterns. Among the tested promoters, *P*_*nasD*_ showed the strongest induction under oxygen-limited conditions, whereas *P*_*narG*_ remained close to baseline and *P*_*srfA*_ displayed intermediate activity. This hierarchy is consistent with the concept that promoters associated with nitrate/nitrite respiration can provide oxygen-dependent self-induction once oxygen becomes limiting and the respiratory program shifts away from oxygen as terminal electron acceptor (Härtig & Jahn 2012). In line with earlier work, *P*_*nasD*_ represents a particularly promising regulatory element for oxygen-responsive expression control and for shifting surfactin formation toward oxygen-limited phases (Hoffmann et al. 2021a).

To generate defined differences in oxygen availability in shake flask screening, the filling volume was varied. This approach is established in lipopeptide-related shake flask studies and is mechanistically linked to reduced oxygen transfer capacity at higher liquid volumes, as reflected in lower volumetric oxygen transfer coefficients and oxygen transfer rates (Fahim et al. 2012; Rangarajan et al. 2015). The observed oxygen-dependent promoter behaviour supports the underlying hypothesis that higher filling volumes lead to earlier and more persistent oxygen limitation and thereby favour induction of nitrate respiration-associated regulation (Hoffmann et al. 2021a). Under these conditions, the transition toward nitrate respiration became apparent from a pronounced increase in pH, which is consistent with the net alkalinisation expected when nitrate is reduced to ammonium as the terminal product of nitrate respiration (Härtig & Jahn 2012; Nakano et al. 1998) (Table S1). The subsequent comparison of MG17 and the promoter-exchange strain MG19 under different filling volumes provides two relevant insights for oxygen switching. First, the online pO_2_ trajectories indicated that oxygen limitation was established in all shake flask cultivations within a comparable time window, and increasing the filling volume did not noticeably accelerate the onset of oxygen-limited conditions. This suggests that under the applied flask geometry and shaking conditions, the oxygen demand of the cultures was sufficient to drive pO_2_ to near zero even at the lowest filling volume of 10% (v/v), thereby diminishing the expected separation of high, moderate, and low oxygen availability based solely on the time point of oxygen depletion. In this situation, filling volume primarily affects the severity and persistence of oxygen limitation rather than its onset, which is consistent with the marked increase in oxygen-based biomass yields (*Y*_X/O2_) when moving from 10 to 50% (v/v). Second, the surfactin profiles and yields confirm that the oxygen-responsive regulation of surfactin synthesis determines whether oxygen limitation can be exploited as a productive trigger. MG17 reached its highest product-to-biomass and product-to-substrate yields at 10% (v/v), whereas MG19 showed reduced surfactin formation under this condition, which is consistent with the intended suppression of surfactin synthesis in oxygen-rich phases. At 25% (v/v), both strains converged to similar surfactin levels and yields, indicating that the oxygen-limited regime became dominant enough to activate the oxygen-responsive production logic in MG19. At 50% (v/v), surfactin accumulation and *Y*_P/X_ decreased for both strains, suggesting that excessively strong and prolonged oxygen limitation becomes restrictive for surfactin formation, likely due to reduced energetic capacity and precursor supply under severe oxygen transfer limitation. This behaviour aligns with the general observation that oxygen limitation can enhance surfactin-related performance within an optimal window, while overly restrictive oxygen availability can reduce overall productivity (Davis et al. 1999; Hoffmann et al. 2021a). The performance indicators further support the interpretation that oxygen limitation altered the balance between growth and product formation rather than overall carbon conversion to biomass *Y*_X/S_ remained in a narrow range across conditions, whereas *Y*_P/X_, *Y*_P/S_, and *q*_P/X_ showed condition-dependent trends that point to an optimum at moderate limitation rather than at the most oxygen-restricted condition. The strong increase in *Y*_X/O2_ at higher filling volumes is consistent with reduced oxygen consumption per formed biomass under oxygen transfer limitation, but it also emphasizes that oxygen-based yields are strongly influenced by oxygen supply constraints and therefore should be interpreted as comparative indicators of oxygen limitation intensity rather than as intrinsic metabolic efficiencies (Heyman et al. 2019; Schiefelbein et al. 2013). Overall, these shake flask results provide a consistent rationale for selecting MG19 for scale-up to the bioreactor and for implementing oxygen switching in a way that establishes micro-aerobic conditions without pushing the system into an overly restrictive regime that compromises surfactin formation.

To translate the filling volume concept into a bioreactor relevant operation mode, fed-batch-like shake flask experiments were subsequently used to emulate a gradual transition from aerobic growth to micro-aerobic conditions under increasing working volume. Varying the interval of the stepwise volume increase between 1 h, 2 h, and 3 h did not produce detectable differences in either the pO_2_ trajectories or biomass formation, indicating that *B. subtilis* MG19 tolerated the tested DO reduction rates without signs of growth collapse. This observation suggests that, within the investigated range, the timing of the oxygen transition is not a dominant stressor at shake flask scale and that the cells can adapt to oxygen limitation independent of the exact reduction rate. This is in line with previous findings that *B. subtilis* can maintain viability during oxygen depletion, although a substantial fraction of cells may lose culturability depending on the physiological state and the severity of oxygen limitation (Arjes et al. 2020). The sampled validation run confirmed that the selected 1 h profile resulted in a reproducible establishment of oxygen-limited conditions and supported surfactin accumulation, while glucose was consumed without major disturbances in growth. The increase in promoter activity toward the end of the cultivation is consistent with oxygen-limited activation of the nitrate-respiration associated regulatory program and supports the intended logic of an oxygen responsive production phase. Taken together, these experiments indicate that stepwise oxygen limitation combined with sequential feeding can be implemented without inducing an apparent loss of biomass at screening scale and provide a practical process blueprint for scale-up. At the same time, the modest final surfactin concentration in shake flasks highlights that transferring the concept to stirred-tank operation is required to exploit tighter control of oxygen supply, feeding, and nitrate availability.

The bioreactor experiments demonstrate that the aerobic to micro-aerobic switching concept is technically implementable at 30-L scale, and that strain architecture critically determines whether switching yields a stable production regime. Across runs A–F, *B. subtilis* MG19 generally tolerated the transition to micro-aerobic conditions without a collapse in cell dry weight, indicating that the promoter-exchange concept can support phase-separated operation at scale. This contrasts with the reference strain *B. subtilis* MG17 in run G, where substantial surfactin formation already occurred during the aerobic phase, followed by a pronounced process disturbance after switching to micro-aerobic conditions. This disturbance was reflected by rapid glucose accumulation and a strong decrease in surfactin concentration, while biomass remained comparatively stable. The magnitude of the surfactin decrease cannot be explained by dilution through feeding alone, since this would require a much larger volume increase than occurred during the process. Instead, the combined pattern of impaired substrate uptake and loss of net surfactin accumulation indicates that MG17 did not maintain a productive metabolic state after the oxygen switch. Such behaviour is consistent with a mismatch between the native regulatory architecture and the imposed oxygen-limited regime, in which the shift in respiratory capacity and uptake kinetics after switching prevents continued substrate conversion and net product accumulation. When benchmarked against state-of-the-art aerobic high cell-density processes, the titers achieved under the present micro-aerobic concept were intentionally not competitive in absolute terms, but they address a different process objective. Fully aerobic fed-batch processes have reached surfactin titers of 26.5 g/L (Klausmann et al. 2021), 36 g/L (Hiller et al. 2024), and 46.33 g/L (Hiller et al. 2025b), enabled by sustained high oxygen transfer and growth rate controlled feeding. Even higher titers have recently been reported by strain-centered engineering approaches, reaching 52.6 g/L through metabolic reprogramming (Gao et al. 2026) and 32.5 g/L through multidimensional strain engineering and peptidoglycan modulation (Xia et al. 2025), further emphasizing that maximum surfactin titers are currently achieved under fully aerobic, high-performance production regimes. These aerobic strategies, however, inherently couple productivity to high aeration and agitation demand and therefore to severe foaming and operational complexity. In the present switching concept, the transition into micro-aerobic operation substantially reduced the need for intensive aeration and agitation during the production phase. Specifically, the aeration rate was reduced from 72 to 18 L/min, corresponding to a 75% decrease, while the agitation speed was reduced from 900 to 300 rpm, corresponding to a 67% decrease. Moreover, no chemical antifoam addition was required during the aerobic phase, highlighting oxygen switching as a practical lever to reduce aeration-related operational constraints in surfactin processes.

The MG19 bioreactor runs further show that oxygen switching must be co-designed with feeding and nitrate supply to avoid imposing a metabolic state that cannot sustain carbon uptake. In the M1 medium runs A and B, glucose accumulation in the late phase indicates that substrate feed exceeded uptake capacity under oxygen-limited nitrate-respiration conditions, even when the feeding growth rate was reduced. In the M2 medium runs C and D, the combination of biomass build-up under aerobic conditions and the subsequent micro-aerobic transition supported higher biomass levels and improved surfactin accumulation relative to runs A and B, with run D representing the best *B. subtilis* MG19 configuration in terms of *P*_max_ and space–time yield. The feed from start strategy by Hiller et al. (2025b) in run E amplified the risk of overfeeding after switching, leading to pronounced glucose accumulation, whereas run F confirmed that reducing the feeding growth rate at the onset of micro-aerobic operation can prevent substrate accumulation, albeit without yielding a substantial improvement in surfactin titer under the applied conditions. Overall, these patterns indicate that the micro-aerobic phase imposes a lower feasible substrate throughput, requiring conservative feeding and robust alignment of electron acceptor availability with the intended production regime.

Comparison to oxygen-limited surfactin control reported previously further emphasizes the importance of controlled reduction of DO and adaptation time. Hoffmann et al. (2021a) demonstrated that oxygen-responsive promoter exchange can shift surfactin synthesis toward oxygen-limited conditions and reported that overly rapid anaerobization in bioreactors can trigger significant cell lysis, whereas gentler transitions using low agitation and aeration show improved stability. The present results are consistent with the general conclusion that switching profiles must provide sufficient physiological adaptation time, but they additionally demonstrate that strain choice can decouple switching stability from the native surfactin regulatory response. MG19 maintained biomass stability after switching in multiple 30 L runs, whereas MG17 exhibited a pronounced collapse in process performance after switching despite stable biomass, indicating that maintaining culturability alone is not sufficient and that net product accumulation can be lost when oxygen limitation disrupts uptake and product balance.

In summary, a thirty-litre bioreactor process enabling an aerobic to micro-aerobic switch was established and a functional separation of growth and production could be realised with *B. subtilis* MG19, while simultaneously reducing aeration-related operational constraints; no antifoam addition was required during the aerobic phase. The current limitation is the low surfactin accumulation under prolonged micro-aerobic operation, suggesting inhibition and/or insufficient metabolic throughput during the production phase. Further intensification is therefore expected to benefit from tighter co-optimization of switching timing, feeding demand, and nitrate supply, combined with in situ handling of foam-active product fractions to maintain a productive micro-aerobic regime (Klausmann et al. 2021; Hiller et al. 2024, 2025b; Hoffmann et al. 2021a).

## Conclusions

The results demonstrate that oxygen availability can be used as a central process control lever to engineer a phase-separated surfactin bioprocess at bioreactor scale. By implementing a defined aerobic growth phase followed by a micro-aerobic production phase, a robust separation of biomass formation and product synthesis was achieved. The engineered strain *B. subtilis* MG19 enabled the intended phenotype, with surfactin formation effectively shifted to oxygen-limited conditions while remaining suppressed during aerobic biomass build-up. In parallel, the transition to micro-aerobic conditions reduced the aeration and agitation demand associated with conventional aerobic surfactin processes. Toward the late cultivation phase, signs of declining process performance became apparent, indicating inhibition or reduced physiological activity under prolonged production conditions. Further process intensification should therefore focus on maintaining micro-aerobic productivity while continuously removing product and foam-active components like acetate from the broth. Integrating an in situ foam-based separation concept such as a foam column is expected to enhance operational stability and sustain surfactin production at elevated titers.

## Supplementary Information

Below is the link to the electronic supplementary material.

ESM 1 (XLSX 3.18 MB)

## Data Availability

The datasets generated for this study are provided in this study and are saved in the Institute of Food Science and Biotechnology, Department of Bioprocess Engineering (150 k), University of Hohenheim, Fruwirthstraße 12, Stuttgart 70599, Germany.
